# Efficacy of Physiotherapy Treatment in Primary Dysmenorrhea: A Systematic Review and Meta-Analysis

**DOI:** 10.3390/ijerph18157832

**Published:** 2021-07-23

**Authors:** Remedios López-Liria, Lucía Torres-Álamo, Francisco A. Vega-Ramírez, Amelia V. García-Luengo, José M. Aguilar-Parra, Rubén Trigueros-Ramos, Patricia Rocamora-Pérez

**Affiliations:** 1 Hum-498 Research Team, Health Research Centre, Department of Nursing, Physiotherapy and Medicine, University of Almería, 04120 Almería, Spain; 2 Hum-498 Research Team, Health Research Centre, University of Almería, 04120 Almería, Spain; lucia.torres.alamo@gmail.com (L.T.-Á.); franavega@hotmail.com (F.A.V.-R.); 3 Distrito Sanitario Poniente, Jesús de Perceval, 22. El Ejido, 04700 Almería, Spain; 4 FQM228-Research Team, Random Models and Design of Experiments, Department of Mathematics, University of Almería, 04120 Almería, Spain; 5 Hum-878 Research Team, Health Research Centre, Department of Psychology, University of Almería, 04120 Almería, Spain; jmaguilar@ual.es (J.M.A.-P.); rtr088@ual.es (R.T.-R.)

**Keywords:** primary dysmenorrhea, pain, physical therapy, electrotherapy, manual therapy

## Abstract

Primary dysmenorrhea (PD) refers to painful cramps before and/or during menstruation. There is a need for emphasis on alternative methods of conservative treatment, so as to reduce the dependence on drugs for alleviating the symptoms. The aim was to find out the effectiveness of some physiotherapy techniques in the treatment of PD. A systematic review and meta-analysis was conducted according to PRISMA standards. The descriptors were “dysmenorrhea”, “physical the-rapy”, “physiotherapy”, and “manual therapy”. The search was performed in five databases: Scopus, PubMed, PEDro, Web of Science, and Medline, in February 2021. The inclusion criteria were randomized controlled trials over the last six years. Articles not related to the treatment of PD or using pharmacology as the main treatment were excluded. Nine articles met the objectives and criteria, with a total of 692 participants. The most used scale to measure pain was the VAS (visual analogue scale). The main techniques were isometric exercises, massage therapy, yoga, electrotherapy, connective tissue manipulation, stretching, kinesio tape, progressive relaxation exercises and aerobic dance. Meta-analysis shows benefits of physiotherapy treatment for pain relief compared with no intervention or placebo (MD: −1.13, 95% CI: −1.61 to −0.64, I^2^: 88%). The current low-quality evidence suggests that physiotherapy may provide a clinically significant reduction in menstrual pain intensity. Given the overall health benefits of physiotherapy and the low risk of side effects reported, women may consider using it, either alone or in conjunction with other therapeutic modalities.

## 1. Introduction

Dysmenorrhea is a menstrual disorder defined by the presence of painful cramps of uterine origin that occur during menstruation. It is one of the most common causes of pelvic pain and short-term absenteeism from school or work, among young and adult women [1,2]. Based on its pathophysiology, dysmenorrhea is classified into two types:

- Primary dysmenorrhea (PD), which is menstrual pain associated with normal ovulatory cycles, without pelvic pathology, and a clear physiological etiology [2]. It is most common in adolescents and young adults.
- Secondary dysmenorrhea, which is menstrual pain associated with an identifiable disease (endometriosis, fibroids, adenomyosis, pelvic adhesions, polyps in the endometrium, pelvic inflammatory disease) or use of an intrauterine contraceptive device [3,4].

The prevalence of PD is highest in the 16–25 year age group but is greatly underestimated as many women consider pain a normal part of the menstrual cycle and do not seek medical treatment, despite the considerable distress they experience [3,5,6]. A previous systematic review on the impact of dysmenorrhea in adolescents reported that the prevalence is high and that it imposes a significant negative impact on academic performance [5], restrictions on daily activities and sports or social and sexual relationships [7]. In terms of incidence, PD decreases with increasing age, similarly affecting all nationalities [3,8,9].

The etiology of PD has been the source of many discussions. Despite research conti-nues on aspects such as its causes and pathophysiology, the theory of prostaglandins (PG) is increasingly consolidated [3,9,10]. Prostaglandins cause narrowing of the blood vessels supplying the uterus, abnormal contractile activity of the uterus, which leads to ischemia, hypoxia of the uterus and increased sensitivity of the nerve endings. It has been demonstrated that prostaglandins are overproduced in dysmenorrhea [10]. PD is characterized by suprapubic colic-type pain that begins a few hours before or after the start of menstrual bleeding. The peak is usually between 24 and 48 h, coinciding with the time of maximum blood flow, and pain usually subsides after 2 or 3 days, as the blood volume decreases. In secondary dysmenorrhea, however, pain might begin before the start of menstrual bleeding and remain after its end (8 to 72 h) [3,11,12,13].

The diagnosis of PD is made by means of an exclusion process, ruling out any organic pathology of gynecological origin [3]. The treatment approach is mainly directed toward relieving the pain through physiological mechanisms that underlie menstrual pain (production of PG). The treatment is also aimed toward the improvement of the function, lea-ding to fewer days lost at work, school or extracurricular activities [1,4,11,14]. 

There are different approaches to the treatment of PD. The drug approach is achieved through PG inhibitors, which are non-steroidal anti-inflammatory drugs (NSAIDs) and hormonal drugs such as contraceptives. Many NSAIDs which non-specifically inhibit both COX-1 and COX-2 enzymes (e.g., ibuprofen) are the most common initial therapy for dysmenorrhea [8,15,16,17,18], but their use is limited by side-effects, such as stomach irritation or ulcer (despite being administered together with gastric protectors) experienced by some women [18]. The prolonged use of NSAIDs is also associated with cardiovascular, hepatic and renal problems [3,18]. Likewise, oral contraceptives are not free from side effects either, related as they are to the frequency of bleeding, weight gain, or the patient’s basal risk of venous thromboembolism [1,19]. All this shows us that there is a need for emphasis on alternative methods of conservative treatment as a non-pharmacological and non-invasive therapy, safe and easy to use for obtaining relief from dysmenorrhea symptoms, including acupuncture and acupressure, biofeedback, heat treatments, transcutaneous electrical nerve stimulation (TENS), exercises and relaxation techniques [20,21]. In addition, one study identifies the need for education on self-care and management of menstrual pain [17].

On the other hand, these physiotherapeutic treatments, being supported by clinical trial data, could be a very useful treatment alternative for women with PD [21], particularly those who are not eligible for pharmacological therapy, since physiotherapy has no side effects according to the analyzed studies [20,21]. Many reviews have evaluated the efficacy of exercise [22,23,24,25] or individual physiotherapy interventions for PD [20,26,27,28]. In 2014, one of these reviews determined the efficacy of physiotherapy modalities in the management of pain [20]. However, the efficacy of physiotherapeutic treatment has not been systematically verified in larger and homogeneous samples in randomized controlled trials and long-term follow-up [3,29]. It is important to search for the most recent evidence for physiotherapy in PD, particularly those studies published since Kannan and Claydon’s systematic review (2014) [20], and also including important outcomes in relation to pain intensity. We have updated the scientific literature to date as new trials have been published for the last six years.

In addition, this systematic review provides practical examples illustrated with photographs (see figures annexed to this article) of isometric exercises, yoga techniques or stretching exercises that women with PD can perform. Therefore, it can be interesting not only for the scientific community or physiotherapists interested in the scientific evidence that the techniques applied in clinical practice provide, but it is also aimed at the women who suffer from this symptomatology since these exercises can be performed autonomously.

The aim of the present study is to describe the effectiveness of different physiothe-rapy techniques in the treatment of PD through a systematic review and a meta-analysis. 

## 2. Materials and Methods

A systematic review was carried out in February 2021, following Preferred Reporting Items for Systematic Reviews and Meta-Analyses (PRISMA) standards [30]. Articles pu-blished in the last six years, whose target population suffered from PD, were procured from various databases, i.e., Scopus, PubMed, PEDro, Web of Science and Medline.

The following PICOS eligibility criteria were used for the selection of the articles (participants, intervention, comparator, outcomes, study design):

Participants were females experiencing PD not using hormonal contraception. Intervention was physiotherapy techniques (electrophysical agents, massage, exercise) and other techniques also used by physiotherapist (yoga, aerobic dance) delivered for at least two menstrual cycles. Comparator was any comparator that did not involve pharmacology. The outcome was pain intensity measured by a validated tool, and regarding the study type, they had to be randomized controlled trials.

The inclusion criteria:

- Contained the following keywords in English “dysmenorrhea”, “physical therapy”, “physiotherapy” and/or “manual therapy. The search strategy used for procuring articles from the different databases is shown in Table 1.
- Randomized controlled trials.
- Physical therapy or conservative treatment techniques listed as the method of intervention.
- Published from 2015 onwards, until 1 February 2021.

The exclusion criteria were:

- Treatment of other pathologies such as endometriosis or dyspareunia.
- Pharmacology as a method of treatment.
- Not written in English or Spanish.

**Figure 1:**
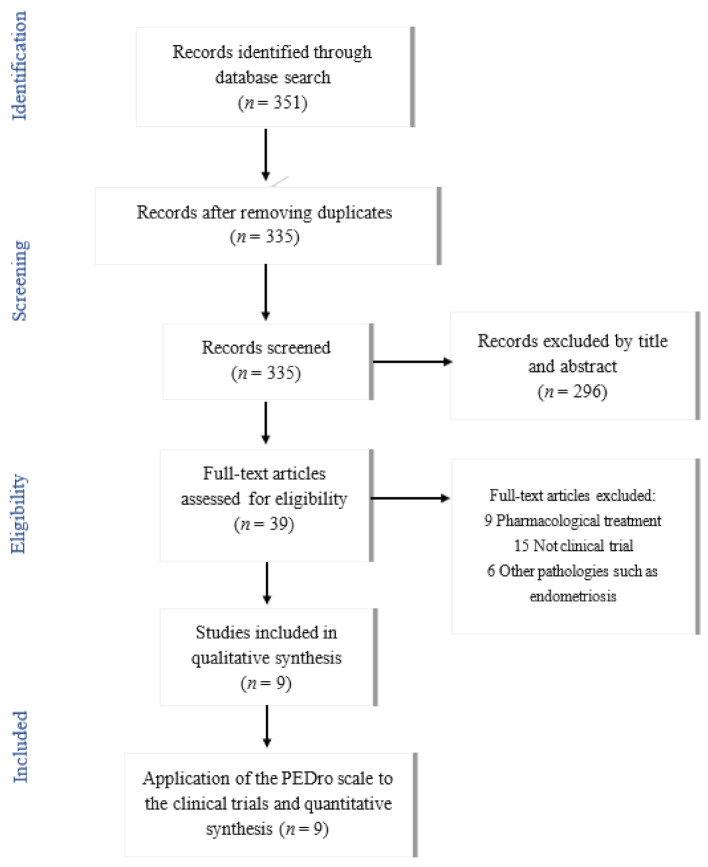
Flowchart of articles selection process.

A total of 351 articles were analyzed based on their title and abstract (Figure 1). These were screened and 296 irrelevant studies were eliminated based on the inclusion criteria. If there was any uncertainty regarding the eligibility of the study from the title and abstract, the full text was retrieved and assessed for eligibility. The search strategy was developed by two researchers (RLL and LTA), who consulted a third researcher if there was any disagreement. After an initial review, 39 articles were considered potentially relevant and an exhaustive reading of their full text was carried out, paying special attention to the intervention (type of treatment), relief of symptoms, number of sessions and duration of treatment. A total of nine articles were finally determined to meet the objective and criteria proposed for this review.

Data analysis were extracted from the intervention details, sample characteristics and variables related to studies results. Two reviewers independently performed study selection, quality assessment and data extraction. Disagreements were resolved by discussion between the reviewers until consensus was reached. Some authors were contacted for any missing data in the included studies.

The Cochrane risk of bias tool was used to assess the included articles, as recommended by the Cochrane Handbook for systematic reviews of interventions [31,32]. The quality of the randomized controlled clinical trials (RCTs) was evaluated using the PEDro scale [33]. This is an 11-item scale designed to help users quickly identify trials that tend to be internally valid (criteria 2–9) and have sufficient statistical information to guide clinical decision-making (criteria 10–11). The score, ranging from 0 to 10, could be determined simply by counting the number of listed criteria that are clearly met in the trial report. Its interpretation is based on the fact that the higher the score, the better is the methodological quality and the lower the risk of bias. The strength of evidence was assessed by the Grading of Recommendations Assessment, Development and Evaluation (GRADE) for menstrual pain intensity, using the GRADE Pro/Guideline Development Tool [34].

A meta-analysis was undertaken using Review Manager software (RevMan version 5.4.1) and limited, owing to the clinical heterogeneity of the included studies. The I^2^ statistic was utilized to determine the degree of heterogeneity, where the percentages quantified the magnitude of heterogeneity: 25% = low, 50% = medium and 75% = high heterogeneity. Using this scale, if I^2^ was 50%, a random effects model was used. All the included outcomes were of data of visual analogue scale (VAS), pain intensity scale (PPI) or numerical rating scale (NRS) and the mean difference with 95% CI was used in analysis. Forest plots were generated to illustrate the overall effect of interventions on pain relief and funnel plots were produced to assess publication bias.

## 3. Results

The search strategy used to identify clinical trials for this review within the databases is described in Table 1. Figure 1 shows the basis for the selection of articles meeting the study objectives and the inclusion/exclusion criteria.

The nine final articles had a total of 692 participants. A summary of the main characteristics of each study is described in Table 2 and Table 3.

In addition, an analysis of the content of the studies has been carried out using the following variables: 

### 3.1. Evaluation or Questionnaires

One of the most important aspects of all the studies was the measurement of pain. The Visual Analogue Scale (VAS, where “0” and “10” represent the minimum and maximum pain level) was used in seven studies [35,36,37,39,40,42,43]. The Present Pain Intensity Scale (PPI, a 0–4 point scale, 0 equal no pain, 4 mean unbearable pain) was used in one of the studies [38], the Numerical Rating Scale (NRS, an 11-point numeric rating scale) [41], and the McGill Pain Questionnaire (Br-MPQ) were also used in one study [41], and the Pain Catastrophizing Scale (PCS, a 13-item instrument, about thoughts or feelings when experiencing menstrual pain on a 5-point scale) also in one study [39]. 

The participants’ anxiety level was also taken into account using the State Trait Anxiety Inventory in three of the studies [35,39,42]. Stress was studied using the Depression, Anxiety and Stress Scale (DASS-21) [39], Pressure Pain Threshold (PPT) and Conditioned Pain Modulation (CPM) [40].

Menstrual symptoms were measured according to their presence and magnitude using the Menstrual Symptom Questionnaire (MSQ) and the Menstrual Attitude Questionnaire (MAQ) [39]. 

Finally, the level of PG2α was measured from blood samples [38].

### 3.2. Interventions or Treatment 

**Figure A1:**
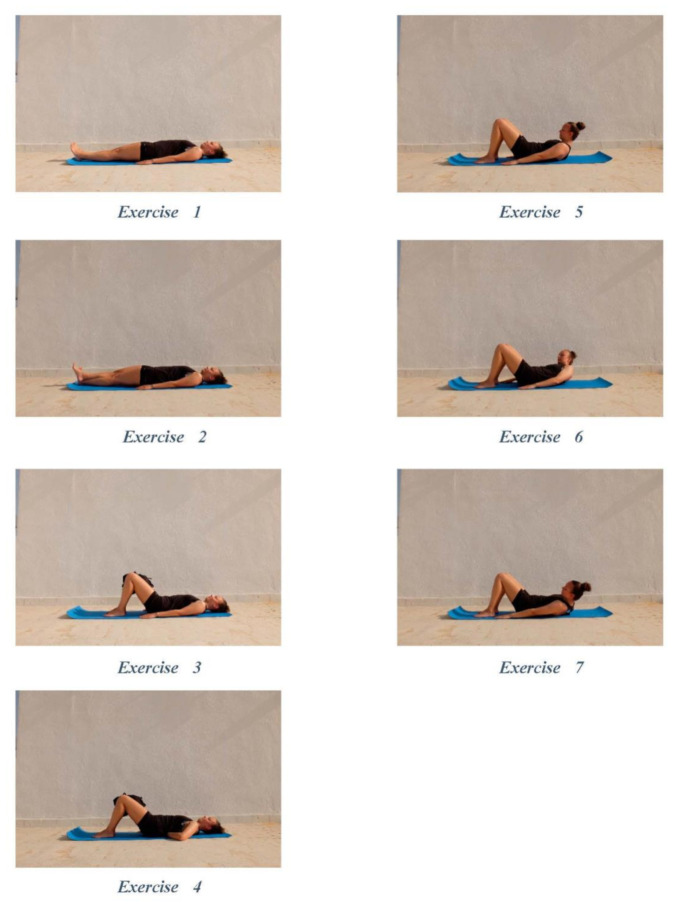
Isometric Exercises [35].

The treatment interventions were diverse in the selected articles. One of them used massage therapy and isometric exercises [35]. The technique chosen was effleurage ma-ssage, performed by applying lavender oil and massaging the area above the pubic symphysis and around the navel, for 15 min, following the direction of the clock. This is a simple, relaxing massage that is carried out with gentle, rotating strokes on the area and is easily tolerated by patients who are in pain. The isometric exercise group had a 7-phase protocol. The seven exercises performed were (Appendix A, Figure A1): 1. Supine position, with legs extended side by side, pressing feet against each other; 2. Supine position, with feet crossed, pressing one against the other; 3. Supine position, knees bent with a pillow between them, pressing the pillow; 4. Same position as in number 3, putting one hand under the waist, pressing against the floor; 5. Supine position, hips and knees bent, lifting head and neck off the floor and holding; 6. Supine position, hips and knees bent, lifting head and neck off the ground turning the head to the right side; 7. Repeat exercise 6, but this time turning to the left side. All exercises were held for five seconds. In addition, deep abdominal breathing was recommended (in supine position, with knees bent, breathing through the nose and directing air into the abdomen) [35].

In another article, a physical therapy program was developed [36], which consisted of 5 phases performed consecutively for 50 min per session. Most of the exercises were performed with 5 to 10 repetitions: 10 min of general stretching; 10 min of specific stretching of the iliopsoas, adductors, and calf muscles; 10 min of jogging at 60–70% of maximum heart rate; 10 min of Kegel exercises to strengthen the pelvic floor muscles; and finally 10 min of relaxation exercises using diaphragmatic breathing techniques [36].

**Figure A2:**
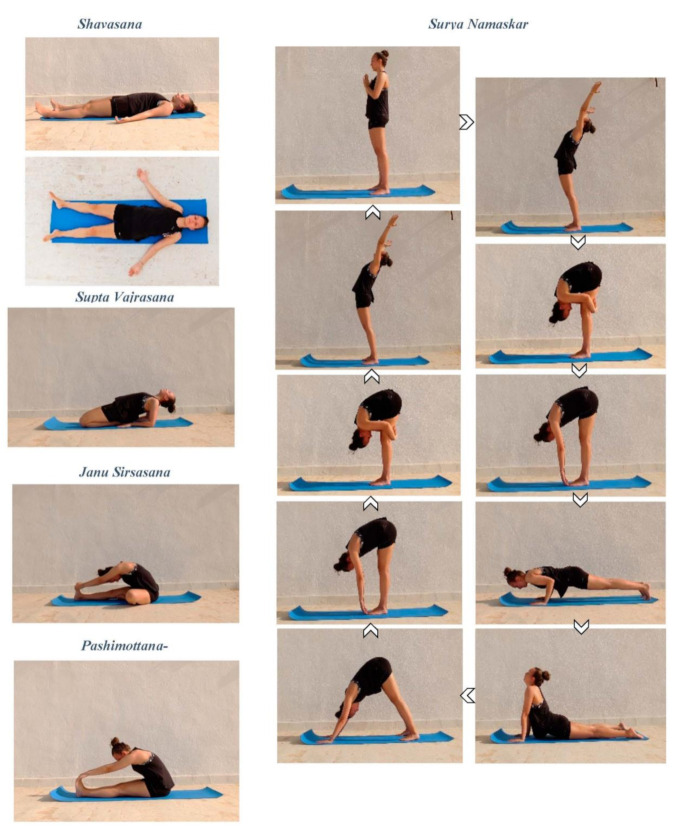
Yoga positions for dysmenorrhea [37].

Yoga was another intervention option [37]. A book named “Yoga for PD” was given to each of the participants. This book included detailed descriptions on how to perform each of the following positions (Appendix A, Figure A2): *Shavasana* (5 min), *Surya Namaskar* (9 min)*, Supta Vajrasana* (2 min)*, Janu Sirsasana* (6 min)*, Pashimottanasana* (3 min)*,* and *Shavasana* (5 min) [37].

Electrotherapy was used in two of the studies [38,41]. One compared High-Intensity Laser Therapy (HILT) to Pulsed Electromagnetic Field (PEMF) [38]. For the HILT group, high levels of energy density (fluidity of 810–1780 MJ/cm), very short pulse duration, between 120–150 µs, a duty cycle of approximately 0.1%, and frequencies of 10–40 Hz were used. Treatment was applied in 3 phases with a total energy of 880 J. During the initial phase, treatment was applied to the suprapubic and paravertebral regions of L4-S3. During the intermediate phase, treatment was applied using a nine-point head, three suprapubic points while the patient was in supine position and six points over the lumbosacral region of L4-S3. The final phase was the same as the initial phase, but with a slow exposure and a total time of 15 min [38]. In the PEMF group, the treatment was applied to the patient in a lateral position. The therapy was applied for 30 min with one electrode on the suprapubic region and another on the lumbosacral region of L4-S3, with a frequency of 50 Hz and an intensity of 60 gauss [38].

In another study [41], the effects of thermotherapy and TENS were compared. Thermotherapy was applied by means of a microwave-type diathermy device. The intensity was set when the patient referred moderate heat sensation. With the patient lying in the supine position, a reflector was directed toward the lower abdomen, kept 5 cm away from the skin surface and held for 20 min. The same unit was used in the placebo group, but the intensity was not adjusted. The application parameters for TENS were: high frequency, in continuous mode, at 100 Hz and 200 µs, with a strong but comfortable intensity according to the sensations of each patient, for 30 min, in the lower abdomen. In the placebo group, TENS was applied with an identical placebo unit. The electrodes in both cases were placed on both sides of the abdomen, at D10-D11 level [41].

Connective Tissue Manipulation (CTM) was applied in one of the studies [39]. The placebo group participants performed stretching exercises, and all participants were given lifestyle advice. In the CTM group, the pelvic regions, including the sacral, lumbar, and lower thoracic areas, as well as the anterior pelvic regions, were manipulated with short and long strokes, respectively. Each stroke was repeated three times, first to the right and then to the left of all the manipulated regions. All sessions ended with bilateral long strokes on the iliac crests and subcostal areas. During the manipulation, the third fingertip was always in direct contact with the patient’s skin. While applying treatment to the back areas, the patient was placed in a seated position, with triple 90° flexion of the lower limbs and feet resting flat. On the other hand, while applying treatment to the anterior pelvic region, the patient was placed in a supine position with pillows under the head and knees [39]. Lifestyle tips given to the patients of both the groups were to exercise regularly, limit caffeine, sugar, and alcohol intake, reduce or quit smoking and avoid exposure to tobacco smoke [39]. Stretching exercises included general stretching exercises to be performed for about 30 min (a total of 6 exercises of quadriceps, calves, both sides of the trunk in bipedal and sitting positions, chest and shoulders), all of them combined with deep abdominal breathing. In addition, a minimum of two and a half hours of moderate-intensity aerobic exercise per week was recommended [39].

Kinesio tape was applied using the ligament technique (75–100% stretch) on the sacral and suprapubic regions in an experimental group compared with a control group and sham tape group (applied on the trochanter major with no tension or technique). Three I-shaped Kinesio tapes with a width of 5 cm and thickness of 0.5 mm were used [42].

Recently, Çelik and Apay [43] conducted progressive relaxation exercises with a CD as the interventional material prepared by the Turkish Psychological Association. First, the researcher performed the exercises and then the students were instructed to do the exercises on their own and were called by phone once a week to remind them to do the exercises regularly. Relaxation exercises start with deep breathing exercises, accompanied by music, and continue with muscle-stretching exercises (tension for 5–7 s, and then loosening the muscles for 15–20 s in the hands, arms, neck, shoulder, face, chest, abdomen, thighs, legs, feet and fingers).

**Figure A3:**
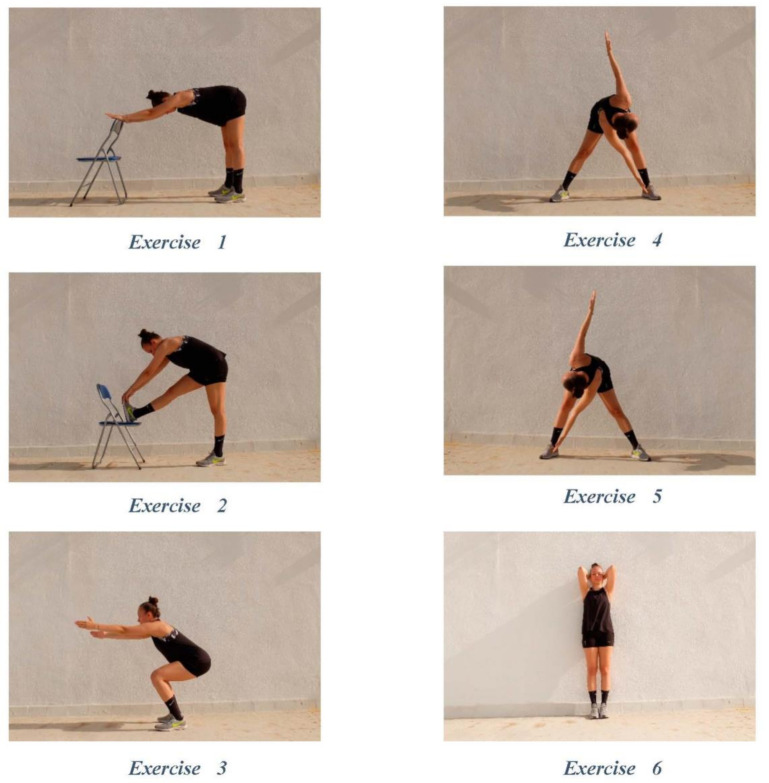
Stretching Exercises [40].

Finally, one of the articles compared stretching exercises to aerobic dance to relieve pain due to PD [40]. Six stretching exercises were recommended (Appendix A, Figure A3): 1. Stand behind a chair, bend the upper part of the body by bending at the hip joint, keeping the back straight and parallel to the floor; 2. Stand about 10–20 cm behind a chair, lift one foot off the floor and place it on the chair to stretch, repeat the same with the other; 3. In standing position, spread the feet to shoulder width, bend the knees and maintain a squatting position; 4. Stand with feet shoulder-width apart and try to touch the left ankle with the right hand, while keeping the head on the mid-line and left hand above the head, then turn the head to look at the left hand. Repeat the same for the opposite side; 5. In supine position, with shoulders, back and feet kept on the ground, the knees are bent with the help of the hands and brought toward the chin; 6. Stand upright, against a wall, placing the hands behind the head with elbows pointing forward. Then, without bending the spine, contract the abdominal muscles [40].

The dance group received aerobic dance 3 days/week for 45 min (10 min warm-up, 25 min dance training, and 10 min cool down). The steps were: walk, one side-slip step, forward and backward steps, two side-slip steps, side step crossing legs, V-step, knee lift, heel to buttock, walk forward, side stride, “L” step and jumping jacks [40].

### 3.3. Methodological Quality of the Included Articles

The methodological quality of the included clinical trials has been evaluated using the PEDro scale [33] (Table 4):

Of the nine articles included in this review, only four of them obtained a score greater than or equal to 7 on the PEDro scale [36,39,41,43]. These are the ones with the highest methodological quality. The remaining articles had a lower score, with the article by Tharani [40] and Thabet [38], scoring the lowest of all. These articles did not meet many of the items on the scale, as no assignments were made, and neither therapists nor assessors were blinded to the study.

**Figure 2:**
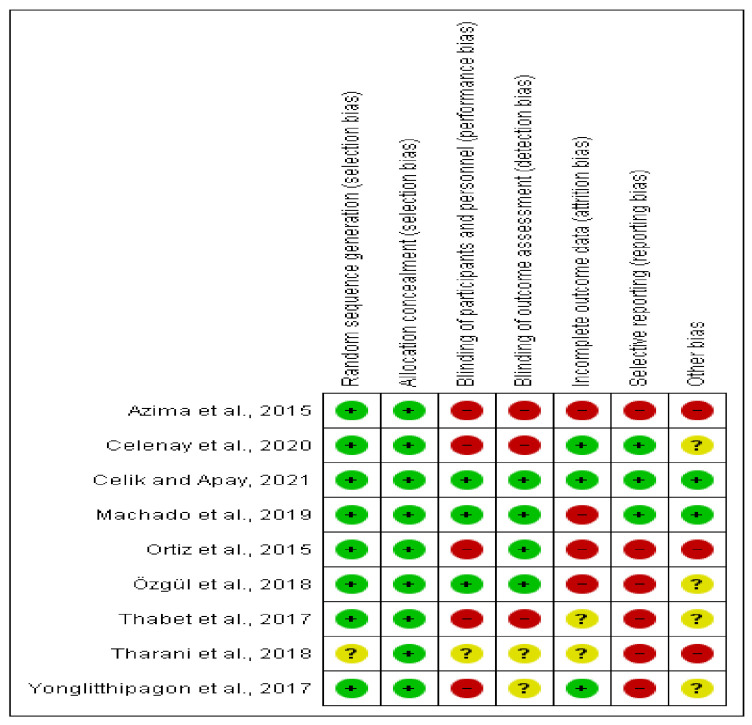
Summary of Risk of Bias in included studies.

Additionally, the Cochrane risk of bias tool [31,32] was used to assess this aspect of the included articles (Figure 2).

Some included studies were at high risk of bias in multiple areas of study design, or they did not report sufficiently so as to reach a conclusion about the risk of bias. The randomization process was described for most studies, except Tharani et al. [40] and allocation concealment was performed in all studies. Due to the nature of the intervention and self-reported outcomes, we rated most trials at high risk of bias in both performance and detection bias. Registered protocol was found in two study [41,42], and we rated them at low risk of reporting bias. Results were sometimes reported incompletely [35]. Some studies reporting follow up loss did not use intention-to-treat analysis [35,36,39,41]. 

### 3.4. Meta-Analysis or Quantitative Analysis of the Included Articles

**Figure 3:**
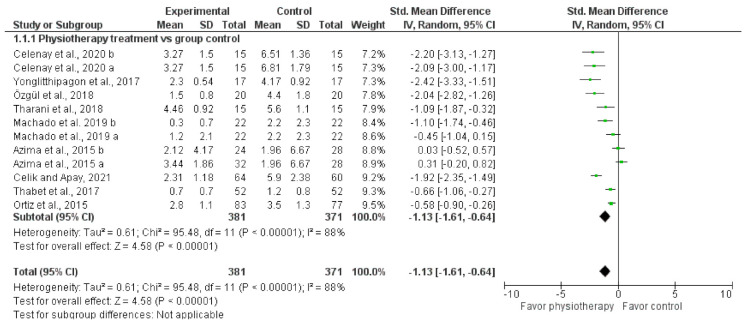
Pooled MD (95% CI) of effect of physiotherapy in pain relief.

Meta-analysis of the nine trials with a total of 692 participants demonstrated that physiotherapy was also better in pain dysmenorrhea than control group (no intervention or placebo) (MD: −1.13, 95% CI: −1.61 to −0.64, I^2^: 88%) (Figure 3).

Table 5 shows the strength of evidence for physiotherapy treatment compared to control group for young women with primary dysmenorrhea.

The quality of evidence was low, the main limitations were inconsistency (studies showed very different results, control groups were clinically heterogeneous) and risk of bias related to blinding (where researchers or participants knew what treatment they were getting). 

The current low-quality evidence suggests that physiotherapy may provide a clinically significant reduction in menstrual pain intensity of around 11 mm on a 100 mm VAS. Given the overall health benefits of physiotherapy and the low risk of side effects reported (0 per 100), women may consider using it, either alone or in conjunction with other therapeutic modalities, to manage menstrual pain.

## 4. Discussion

This review describes various conservative alternatives for the treatment of PD in light of clinical trials carried out over the last six years. The aim of this systematic review has been to find out the effectiveness of some physiotherapy techniques in the treatment of PD: isometric exercises, massage therapy, yoga, electrotherapy, connective tissue manipulation, stretching, kinesio tape, progressive relaxation exercises and aerobic dance. Meta-analysis has shown benefits of physiotherapy treatment for pain relief compared with no intervention or placebo. Physiotherapy techniques can be considered as potential alternatives to analgesic medication. However, difficulties in controlling for non-specific effects, along with potential for bias, may influence study findings.

PD is described in the scientific literature as one of the most frequent dysfunctions in gynecological consultations [44]. It is also cited as one of the most common reasons behind short-term school or work absenteeism among young and adult women [1]. The treatment of PD is mainly aimed toward relieving the pain and other associated symptoms (as back and leg pain, anxiety, stress and other symptoms that affect quality of life [37,39,40,42]. Most women opt for drug treatment to alleviate the symptoms despite its many side effects [10,17,21]. 

As described by García et al. [3], Kannan and Claydon [20], or Corral-Moreno et al. [45], physiotherapy can be a very effective treatment for PD, offering different alternatives, and with the advantage that it can sometimes be performed autonomously by the patient. There are authors who also recommend treatment through electrotherapy [26,38,41,45], and others who recommend the use of manual therapy [35,39,45], acupressure [28,45,46], Kinesio tape [42,47,48], progressive relaxation exercises [43] or therapeutic exercise [22,24,49,50].

Machado et al. [41], suggested that thermotherapy and TENS can serve as good options of treatment, highlighting in their article the value of thermotherapy for symptom reduction. The electrotherapy modality was already used for the treatment of PD by Vance et al. [51], who, being the pioneers in applying microwave-type diathermy, began to describe its effectiveness and suggested its comparison with TENS. The effectiveness of TENS therapy has also been supported by other authors such as Wang et al. [52], Arik et al. [26], or Tugay et al. [53]. These last mentioned authors compared the use of TENS and interferential currents, concluding that both the treatments are effective in reducing symptoms.

According to Thabet et al. [38], PD can be treated with High Intensity Laser Therapy (HILT) and Pulsed Electromagnetic Field (PEMF), the former being more effective in reducing pain and blood PG levels. This type of intervention was supported by Shin et al. [ 54], who reaffirmed the effectiveness of laser therapy in the symptomatic relief of PD, and suggested that pain caused by abnormal functioning of the smooth muscles of the uterus can be treated by means of this therapy [54]. Therefore, all the studies mentioned here [38,41,52,53,54] considered electrotherapy to be a useful therapeutic option for reducing the pain and other symptoms of PD, with the advantage of having no side effects. 

Authors such as Azima et al. [35], chose to propose aromatic massage as an intervention method, in concordance with the study carried out by Apay et al. [55]. Both defended the effectiveness of massage therapy for pain relief and, if it is done with aromatic oils, the benefit is greater. It is easy to apply, safe, low cost and without side effects.

According to Özgül et al. [39], connective tissue manipulation is an effective method that can be adopted for short-term pain relief. This study, in conjunction with the observational pilot study previously conducted by Reis et al. [56], showed improvement through this therapy. However, both the studies pointed out the need for more randomized, placebo-controlled studies to confirm the results. These studies also advocated long-term follow-up to test whether it is possible to achieve full remission of symptoms or if it is only effective for pain relief [39,56].

Authors Celenay et al. [42], Boguszewki [47] and Hanife [48] agree on KT application appearing to be an effective method in decreasing pain, anxiety level and some menstrual complaints such as abdominal and leg pain, fatigue, vomiting, diarrhea and nausea, as well as in reducing medication use. KT is an effective, easy and complementary tool for reducing symptoms in PD and improving quality of life and body awareness [48]. However, further studies using objective investigative tools are needed (e.g., the measurement of underbelly muscle tone or thermography). 

Several studies included in this review proposed guided exercise treatments [22,36,37,40] or progressive relaxation exercises [43] that could be performed autonomously by the patients. Four studies conducted by Azima [35], Ortiz [36], Tharani [40] and Yonglitthipagon et al. [37], applied a physical therapy program for PD (isometric exercises, stretching exercises, aerobic dance or yoga, respectively). They all opted for dynamic treatments, showing positive results in improving the symptoms and quality of life of sedentary women, thereby aiding the prevention of numerous other complications that may arise due to the lack of physical exercise. Similarly, Carroquino et al. [22] conclude in their systematic review that the most effective exercise programs were stretching and isometric exercises for 8 weeks for pain intensity and duration, yoga for 12 weeks for pain intensity and quality of life and aerobic exercises for 12 weeks for quality of life. Gotpagar and Devi [49] study the effect of Bosu Pilates (exercises like stretching and core strengthening exercises performed on bosu ball) which helped to reduce pain on PD.

However, the effectiveness of these programs in women who regularly practice sports is unclear. There is heterogeneity with respect to the the way to apply exercise for dysmenorrhea [23]. In the Cochrane review conducted by Armour et al., the available evidence supporting the use of exercise to treat PD was examined and concluded to be low-quality evidence suggesting that exercise, performed for about 45 to 60 min each time, three times per week or more, may provide a clinically significant reduction in menstrual pain intensity of around 25 mm on a 100 mm VAS [23].

In Kim’s meta-analysis [24] it was concluded that yoga is an effective intervention for alleviating menstrual pain in women. Kirmizigil and Demiralp [50] confirm the positive effects of a regular and combined exercise program, which reduces pain severity in the low back and abdomen, and other menstrual symptoms, and improves sleep quality pain. For its part, progressive relaxation exercises, performed on a regular basis, have an impact on improving immune function, reducing depression and enhancing daily life; Çelik and Apay suggest the usage of these relaxation exercises to decrease dysmenorrhea pain and for analgesic use [43].

Sharghi´s review [27] included 17 papers, 10 of which on complementary medicine (medicinal plants), three on drug therapies, and four on acupuncture and acupressure. Further trials are required to confirm the benefits of the procedures described and ensure the absence of complications. Kannan and Claydon´s review [20] identified that heat, TENS, and yoga can each significantly reduce the pain of dysmenorrhoea. The 11 included trials compared intervention as TENS, spinal manipulation, continuous low-level heat, yoga, acupuncture and acupressure. Although acupuncture and acupressure reduced pain severity in dysmenorrhea, this appears to be a placebo effect. Kannan and Claydon´s data confirmed similar results for the physiotherapy techniques we have considered, including isometric exercises, massage therapy, yoga, electrotherapy, connective tissue manipulation or stretching. Given that the costs and risks of these interventions are low, they could be considered for clinical use.

The studies included in this review used various scales, such as the VAS scale [35,36,37,39,40,42,43], the NRS scale [41] and the PPI scale [38] to assess pain, which itself is the main symptom in dysmenorrhea. These trials reported data suitable to be included in the meta-analysis although further research is required, using validated outcome measures, adequate blinding and suitable comparator groups reflecting current best practice.

One of the limitations of the present study is the use of a small sample size in some of the studies included in the analysis. Another drawback is that none of the studies selected for this review applied the treatment for longer than three months, i.e., three menstrual cycles. Many other keywords or MESH terms related to “physiotherapy topic” could have been included in the search strategy; thus, some studies might have been missed. Likewise for databases as EMBASE or CINAHL. We are aware that some literature on physiotherapy may be excluded from the main databases: grey literature sources or studies identified in other different languages than English or Spanish were not considered. A high I^2^ statistic suggests that variations in effect estimates may be due to differences between trials, because studies evaluated a wide range of physiotherapy interventions. Finally, the risk of bias was unclear for many domains in most of the included studies.

Therefore, studies with a larger sample size are required. Future research should be carried out over longer periods to measure long-term outcomes. The results of the present analysis suggest that in order to improve the quality of life of women suffering from PD, new clinical trials with a physiotherapy protocol for dysmenorrhea should be carried out in the future, keeping in view the limitations of the current studies. This would ensure high quality studies to be carried out on treatments aimed toward achieving the reduction of symptoms in as many women as possible.

The practical implications of this analysis could be a reduction in the use of pharmacological treatment by using physiotherapy as a treatment alternative. This would, in turn, lead to less side effects and a reduction in the financial expenditure on NSAIDs, contraceptive pills or any other drugs that are commonly used by PD patients. Physiotherapy offers a varied, sufficient arsenal of techniques that could be applied in an individualized way to each patient, so as to reduce the secondary effects after its application, and thereby improving women’s quality of life. The need and importance of educating patients to consider PD as something that can and should be treated must be stressed. It is a matter of importance to free women from their position of acceptance and conformity in regards to this issue, and to encourage them to seek solutions that, until now, have been kept unknown to a great majority. Given the overall health benefits of exercise, and the reported relatively low risk of side effects of physiotherapy in the general population, women may consider using these conservative treatments. But not everything that might be done will be beneficial, independently of what and how it is performed, it is necessary for a physiotherapist to supervise the adequacy and correct performance of the selected techniques, individually. For example, the mere use of pictures and written explanation may be misleading if one does not receive a face-to-face explanation on how to perform a given exercise accurately, which could lead to malperformance and, as a result, decrease in any effectiveness that this exercise may have.

## 5. Conclusions

The current low-quality evidence suggests that conservative treatments, such as certain physiotherapy techniques, may provide clinically significant symptoms reduction with the advantage of no side effects.

## Figures and Tables

**Table 1:** Search strategies in the different databases.

Databases and Search Terms	Results	Selected Articles
SCOPUS		
1. “Physical therapy” AND “dysmenorrhea”	50	16
2. “Physiotherapy” AND “dysmenorrhea”	115	
3. “manual therapy” AND “dysmenorrhea”	14	
PUBMED		10
1. “Physical therapy” AND “dysmenorrhea”	38	
2. “Physiotherapy” AND “dysmenorrhea”	43	
3. “manual therapy” AND “dysmenorrhea”	3	
PEDRO		5
1. “Physical therapy” AND “dysmenorrhea”	5	
2. “Physiotherapy” AND “dysmenorrhea”	11	
3. “manual therapy” AND “dysmenorrhea”	2	
WEB OF SCIENCE		4
1. “Physical therapy” AND “dysmenorrhea”	26	
2. “Physiotherapy” AND “dysmenorrhea”	11	
3. “manual therapy” AND “dysmenorrhea”	3	
MEDLINE		4
1. “Physical therapy” AND “dysmenorrhea”	16	
2. “Physiotherapy” AND “dysmenorrhea”	11	
3. “manual therapy” AND “dysmenorrhea”	3	

**Table 2:** Characteristics of the study and the participants.

Author, Year	Type of Study	Sample Size (Participants)	Age	Measured Variables	Ain Results
Azima et al. [35] 2015	Randomized clinical trial	102 participants 34: EG 1 34: EG 2 34: CG	19–23 years old	Pain (VAS), duration of pain (hours) and anxiety (STAI).	Significant improvement in pain intensity in both EGs (massage EG1 and isometric EG2), but greater in massage therapy group (*p* < 0.001) in the 2nd and 3rd cycle.
Ortiz et al. [36] 2015	Prospective, parallel-group, randomized clinical trial	173 participants 89: EG 84: CG	18–22 years old	VAS, presence and magnitude of symptoms (LS).	Significant reduction of pain in EG according to VAS from the 2nd and 3rd menstrual cycles (*p* < 0.05) compared with the CG.
Yonglitthipagon et al. [37] 2017	Randomized clinical trial	34 participants 17: EG 17: CG	18–22 years old	VAS; quality of life (SF-36); flexibility (SR), and back and leg strength (dynamometer).	Statistically significant differences in yoga EG in terms of pain intensity, flexibility, and muscle strength (*p* < 0.02).
Thabet et al. [38] 2017	Randomized clinical trial	52 participants 26: EG 1 26: EG 2	18–24 years old	Pain Intensity was measured with PPI (0–4 scale); pain relief scale; and prostaglandin PG2α concentration with blood samples.	Both groups had a decrease in pain, but the effect was more pronounced in the HILT group (*p* < 0.05). There was a decrease in the PG2α level in both groups (*p* < 0.001).
Özgül et al. [39] 2018	Randomized controlled clinical trial	44 participants 21: EG 23: CG	aged over 18 years old	Pain intensity (VAS and PCS), anxiety level (STAI), menstrual symptoms (MSQ) and menstrual attitude (MAQ).	CTM group showed statistically significant improvement in pain, medication use, PCS, MSQ (*p* = 0.001) and in the perception of menstruation (*p* = 0.029).
Tharani et al. [40] 2018	Pre- and post-comparative experimental study	30 participants 15: EG 1 15: EG 2	17–23 years old	Stress (DASS-21) and pain (VAS).	Both groups showed a reduction in pain and stress, but aerobic dance was significantly more efficient (*p* < 0.001).
Machado et al. [41] 2019	Placebo- controlled, double-blind clinical trial	88 participants 22: EG 1 22: EG 2 22: EG 3 22: CG	18–44 years old	Pain intensity (NRS, Br-MPQ), pressure pain threshold (PPT) and conditioned pain modulation (CPM).	Thermotherapy reduced pain intensity compared to TENS (*p* = 0.01) and placebo (*p* = 0.05) after 20, 110 min, and 24 h.
Celenay et al. [42] 2020	A randomized sham- controlled trial	45 participants 15: EG1 15: EG2 15: CG	18–35 years old	VAS, anxiety level (STAI), and menstrual complaints.	The decreases in pain, anxiety levels, and menstrual complaints were higher in the KT group than those in the other two groups (*p* < 0.05).
Çelik and Apay [43] 2021	A randomized prospective controlled trial	124 participants 64: EG 60: CG	18–22 years old	VAS and a dysmenorrhea monitoring form.	Progressive relaxation exercises are an effective method for reducing PD.

EG: Experimental Group; CG: Control Group; PD: Primary Dysmenorrhea; VAS: Visual Analogue Scale; STAI: State Trait Anxiety Inventory; LS: Likert Scale; SR: Sit and Reach test; HILT: High Intensity Laser Therapy; PPI: Present Pain Intensity scale; CTM: Connective Tissue Manipulation; PCS: Pain Catastrophizing Scale; MSQ: Menstrual Symptom Questionnaire; MAQ: Menstrual Attitude Questionnaire; DASS-21: Depression, Anxiety and Stress Scale; TENS: Transcutaneous Electrical Nerve Stimulation; PPT: Pressure Pain Threshold; CPM: Conditioned Pain Modulation; NRS: Numerical Rating Scale; Br–MPQ: McGill Pain Questionnaire; KT: kinesio tape.

**Table 3:** Characteristics of the intervention of the selected studies.

Author, Year	Description INTERVENTION	Duration of Sessions	Follow-Up	Support
Azima et al. [35] 2015	Effect of massage therapy and isometric exercises on PD. EG 1. Massage therapy: effleurage massage in the pubic symphysis and navel area with lavender oil. EG 2. Isometric exercises. These exercises included 7 stages. CG. No intervention.	EG1: two consecutive cycles of effleurage massage (15 min each). On the first day of onset of menstrual pain and the following day. EG2: from 3rd day of menstruation for 8 weeks, 5 days/week, 2 sessions/ day, and 10 times/session.	Pain intensity and duration were measured in 3 consecutive cycles (at the peak of PD). The level of anxiety was measured 4 and 8 weeks after the treatment.	Exercises modified and confirmed by a specialized rehabilitation consultant.
Ortiz et al. [36] 2015	Effectiveness of physical therapy treatment in relieving PD. EG. Physiotherapy program: Stretching, specific stretches, Kegel exercises, jogging, and relaxation exercises, performed 5–10 repetitions. CG. No intervention.	5-phases of the physiotherapy program, for 50 min, 3 times/week, for 3 menstrual cycles (three months).	Measurements within 5 days after the last day of menstruation.	The program was led and monitored by three trained researchers.
Yonglitthipagon et al. [37] 2017	Effect of a yoga program designed for non-athletic women with PD. EG Yoga. Poses of Shavasana, Surya Namaskar, Supta Vajrasana, Janu Sirsasana and Pashimottanasana GC. No intervention.	EG yoga for 30 min. per day, twice a week, for 12 weeks at home.	Evaluation at the beginning and at 12 weeks.	The booklet “Yoga for PD” was given to participants.
Thabet et al. [38] 2017	Efficacy HILT compared to PEMF. EG 1. High Intensity Laser Therapy group. 15 min./ session EG 2. Pulsed Electromagnetic Field group. 30 min./ session.	3 sessions, 3 consecutive menstrual cycles, when pain was intolerable (1st and 2nd day of menstrual flow).	Pain was assessed before and after the treatment, along with its relief after each treatment, and blood samples were collected before and after 3 months.	The devices were calibrated at the Department of physical therapy.
Özgül et al. [39] 2018	Short-term effectiveness of Connective Tissue Manipulation (CTM). EG. CTM in pelvic zones (sacral, lumbar, lower thoracic) and anterior pelvic regions.CG. Lifestyle tips and stretching exercises.	The EG received CTM for 5 days/week, from ovulation until the beginning of the next period.	Measurements were taken at the beginning and immediately after the first menstruation in postintervention period.	Treatment was performed by a trained physiotherapist.
Tharani et al. [40] 2018	Effects of stretching exercises in PD compared to aerobic dance. EG 1. Stretching and advice. EG 2. Aerobic dance. Both avoided exercise during the menstrual cycle.	Stretching EG1 and aerobic dance EG2, 45 min. for 3 days/week, for 8 weeks.	The evaluation was performed before and after the treatment.	Carried out in the Faculty of Physiotherapy.
Machado et al. [41] 2019	Effects of thermotherapy and TENS, PPT and CTM in women with PD. EG1. Thermotherapy and TENS. EG2. Thermotherapy (microwave diathermy, 20 min.) EG3. TENS (200 μs, 100 Hz, 30 mi) CG. Placebo	One session Thermotherapy 20 min. TENS 30 min.	The evaluation was performed: at the beginning, after 20, 50 and 110 min., and 24 h. after the intervention.	Physiotherapy Department
Celenay et al. [42] 2020	Effects of kinesio tape (KT) application on pain, anxiety, and menstrual complaints in women with PD. EG1. Kinesio Tape EG2. Sham Tape CG. No tape application	KT 2 days a week, from the ovulation until the next period begins.	Before and after the applications.	Applied by an experienced physical therapist (Kenzo Kase’s Kinesio Taping Method).
Çelik and Apay [43] 2021	Effects of progressive relaxation exercises on pain in PD. EG. Progressive relaxation exercises CG. No intervention	Exercises, average of 30 min. for two months, every day or at least three times a week.	Values measured at the first, second, and third cycles.	Exercises were self-administered via compact disc.

EG: Experimental Group; CG: Control Group; PD: Primary Dysmenorrhea; HILT: High Intensity Laser Therapy; PEMF: Pulsed Electromagnetic Field; CTM: Connective Tissue Manipulation; TENS: Transcutaneous Electrical Nerve Stimulation; KT: Kinesio tape; min: minutes; h: hour.

**Table 4:** Summary of the quality of the randomized clinical trials included in this review based on the PEDro scale.

Item (PEDro Scale)	1	2	3	4	5	6	7	8	9	10	11	Total Score
Azima et al. [35] 2015	X	X	N	N	N	N	N	X	N	X	X	5
Ortiz et al. [36] 2015	X	X	X	X	N	N	X	N	X	X	N	7
Yonglitthipagon et al. [37] 2017	N	X	X	X	N	N	N	N	N	X	X	5
Thabet et al. [38] 2017	N	X	N	X	N	N	N	N	N	X	X	4
Özgül et al. [39] 2018	X	X	X	X	N	N	X	X	N	X	X	8
Tharani et al. [40] 2018	N	X	N	X	N	N	N	N	N	X	X	4
Machado et al. [41] 2019	N	X	X	X	X	N	X	N	N	X	X	7
Celenay et al. [42] 2020	N	X	N	X	N	N	N	X	N	X	X	5
Çelik and Apay [43] 2021	X	X	X	X	X	N	N	N	N	X	X	7

N: the criterion is not satisfied; X: the criterion is satisfied.

**Table 5:** Grading of Recommendations Assessment, Development and Evaluation (GRADE) evidence profile.

Certainty Assessment							No of Patients		Effect		Certainty	Importance
No of Studies	Study Design	Risk of Bias	Inconsistency	Indirectness	Imprecision	Other Considerations	Physiotherapy Treatment	Control Group	Relative (95% CI)	Absolute (95% CI)		
9	randomised trials	serious ^a^	serious ^b^	not serious	not serious	none	381	371	-	MD 1.13 lower (1.61 lower to 0.64 lower)	⨁⨁◯◯ LOW	IMPORTANT

Question: Physiotherapy treatment compared to control group for young women with primary dysmenorrhea. Outcome (assessed with: VAS, PPI, NRS) CI: Confidence interval; Menstrual pain intensity, measured by various outcome measures including VAS (visual analogue scale), PPI (present pain intensity scale) and NRS (numeric rating scale) at the end of the intervention. Lower scores indicate less intense menstrual pain. ^a^ Downgraded one level for serious risk of bias: all included trials are at a high risk of bias for performance and detection bias. ^b^ Downgraded one level for serious inconsistency: while heterogeneity is very high (I2 = 88%), all effects are in the same direction, favouring physiotherapy.
